# Improving the Accuracy of a Biohybrid for Environmental Monitoring

**DOI:** 10.3390/s23052722

**Published:** 2023-03-02

**Authors:** Michael Vogrin, Wiktoria Rajewicz, Thomas Schmickl, Ronald Thenius

**Affiliations:** 1 Institute of Biology, University of Graz, 8010 Graz, Austria; 2 Institute of Psychology, University of Graz, 8010 Graz, Austria

**Keywords:** biohybrid, environmental monitoring, biosensor, signal detection, judgment and decision making, sustainable environmental monitoring technology

## Abstract

Environmental monitoring should be minimally disruptive to the ecosystems that it is embedded in. Therefore, the project Robocoenosis suggests using biohybrids that blend into ecosystems and use life forms as sensors. However, such a biohybrid has limitations regarding memory—as well as power—capacities, and can only sample a limited number of organisms. We model the biohybrid and study the degree of accuracy that can be achieved by using a limited sample. Importantly, we consider potential misclassification errors (false positives and false negatives) that lower accuracy. We suggest the method of using two algorithms and pooling their estimations as a possible way of increasing the accuracy of the biohybrid. We show in simulation that a biohybrid could improve the accuracy of its diagnosis by doing so. The model suggests that for the estimation of the population rate of spinning *Daphnia*, two suboptimal algorithms for spinning detection outperform one qualitatively better algorithm. Further, the method of combining two estimations reduces the number of false negatives reported by the biohybrid, which we consider important in the context of detecting environmental catastrophes. Our method could improve environmental modeling in and outside of projects such as Robocoenosis and may find use in other fields.

## 1. Introduction

The water-quality crisis has been a subject of interest for many years. Farming, air pollution, and other anthropogenic factors have contributed to the rapid decay of natural habitats [1]. Extensive monitoring of the water quality is considered crucial to taking preventative measures against further diminishing of freshwater supplies. Traditionally, data collection on the water quality has been carried out using sampling and classical sensors (e.g., oxygen probe) [2,3,4]. While these methods are highly precise and reliable, they pose a significant challenge for long-term, extensive monitoring. Sampling techniques are often time- and money-consuming and do not provide real-time data. Thus, it is relatively difficult to obtain a good representation of sudden and quick events occurring in the environment. Traditional probing methods can provide continuous data on a selected number of water parameters. However, due to the complexity of aquatic ecosystems, monitoring the handful of abiotic factors might not be sufficient to accurately estimate the state of the environment [5]. Further, the use of specific sensors for environmental monitoring presupposes knowledge about which factors and substances are relevant. Many biotic factors, such as changes in species distribution, toxins, and diseases for which there are no sensors, affect the aquatic communities and the environments they inhabit [6].

Automated systems that combine recent technological developments with living organisms in so-called “biohybrids”, can achieve continuous and more robust water monitoring [7]. Biohybrids are devices that combine artificial components, such as electronics and mechanical parts, with biological parts. The methodology of the EU-project Robocoenosis [8] is to use biohybrids to observe organisms, which are adapted to their respective environments and are thus likely to react to changes occurring in them. By reading those reactions, a different type of data can be obtained compared to the traditional monitoring methods. An early-warning system based on behavioral data is more robust and can provide information on the combined effects of changes affecting the water body of interest.

The concept of using organisms as living sensors has been investigated and implemented in the past. Organisms often react to their environments in predictable as well as measurable ways, and can be understood as bioindicators. For example, a macrophyte *Typha* sp. has been recognized as a good bioindicator for cadmium (Cd) and nickel (Ni) due to the accumulation of these components in the leaves [9]. In the animal kingdom, mussels have been used in automatic monitoring systems under laboratory conditions as well as directly in the field. A device that reads reactions from bioindicators is a biosensor. For example, Mosselmonitor^®^ is a machine that monitors the valve movements of a bivalve *Mytilus galloprovincialis*. A disruption in normal behavior triggers an alarm and indicates the need for further investigation [10]. Recently, a freshwater ostracod (*Heterocypris incongruens*) was considered for biohybrid sensors [11].

Another organism frequently used as a bioindicator is *Daphnia* sp. (Müller, 1785). This cladoceran is an important part of the aquatic food web and its biology and ecology have been extensively researched [12,13]. Thanks to its sensitivity to many toxic compounds such as pesticides, crude oil, heavy metals, and others, it has become a popular choice for ecotoxicological assessments [14,15]. Their stress responses include movement inhibition, spinning (erratic swimming), increased sinking, increased heart rate, disruption in phototaxis, and many others [16,17,18]. For the purposes of this study, we focus specifically on the monitoring of *Daphnia* and the automatic detection of its spinning behavior as an example case. To summarize, *Daphnia* serve as bioindicators and the module that detects the spinning *Daphnia* serves as a biosensor.

The idea of the proposed biohybrid is to have a small sample population within an observational chamber. By observing *Daphnias*’ well-being, indicated by their swimming pattern, we can gain insight into the overall state of the environment [8]. Additionally, by reading the intensity and type of the stress reaction, potential causes of disruption can be identified or narrowed down. By using the representative population enclosed in the biohybrid’s observational chamber, it is possible to extrapolate and draw conclusions on the population’s immediate surrounding. In case a threshold is exceeded (i.e., when enough individuals present a disrupted behavior, such as spinning), an alarm is triggered which indicates the need for further investigation of the body of water.

**Figure 1:**
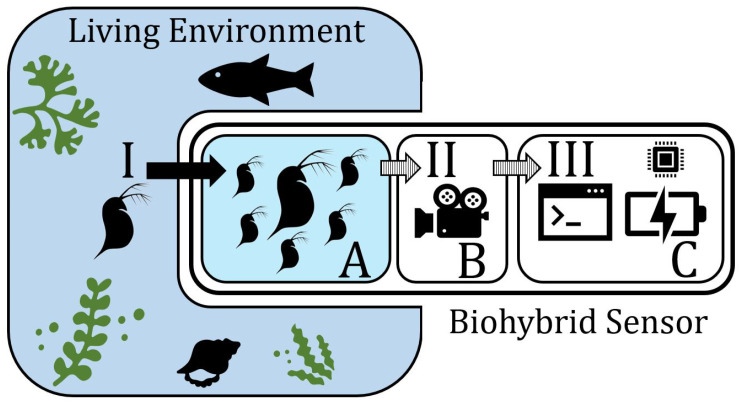
The module relevant for *Daphnia* observation consists of three parts that are shown in this schematic. There is an inflow (I) of water, nutrients, and potential pathogens from the living environment into the observational chamber (A). This chamber houses a sample population of living *Daphnia* and their swimming behavior is recorded (II) by a camera module (B). The recordings are the input (III) for the image-detection algorithm, which detects abnormal swimming behavior as a signal for environmental stressors, and is run in the remaining part of the module, where processors and energy sources reside (C).

The biohybrid module designed for *Daphnia* is part of a larger biohybrid entity. The monitoring setup is placed in the desired location where it begins the observation of the animals and gathers data on their behavior. The setup consists of the main robotics tube, containing electronic parts (camera, power supply, and a Raspberry Pi), as well as a *Daphnia* flow-through chamber and background lighting. Due to the transparency of the body of *Daphnia*, the background illumination is necessary for the camera to be able to pick up the animals and analyze their movements. The flow-through cage is constructed with mesh elements to allow for the surrounding water of the environment and its respective ecosystem to flow through the chamber and provide the *Daphnia* with nutrients from the environment. By this, *Daphnia* is in dense contact with the ecosystem. An algorithm developed for *Daphnia* tracking uses image analysis to extract their trajectories and identify potential unusual behaviors. These unusual behaviors are signals that indicate that the *Daphnia* are stressed due to environmental conditions. When the threshold is crossed (for example, when enough individuals show disrupted behavior) the biohybrid can detect it and take actions (relocate, sound an alarm, increase the sampling rate, etc.). We show this setup schematically in Figure 1.

Since the biohybrid will operate most of its lifetime autonomously, the observation of the sample population of *Daphnia* will be automated using an image analysis algorithm. Notably, the possibility of noise is significantly greater than above water, due to air bubbles, floating algae particles, dust, sediments, and other lifeforms entering the observation chamber. Further, images might be blurred because of the scattering of light by the water and its constituents [19]. These factors make image analysis challenging in underwater setups, creating challenges when trying to identify the exact number of *Daphnia* that are spinning in the chamber at all times. Consequently, said noise can lead to false-positive results in cases where particles are swirled by the water movement, often in a circular motion, resembling the spinning *Daphnia*. On the other hand, false-negative results are possible when *Daphnia* spin on an axis that is not captured well by the camera and are then not identified as spinning. Because of such difficulties, an estimation algorithm must be developed to improve the accuracy of the estimation of the number of spinning *Daphnia* within the setup.

The biohybrid has important limitations in terms of number of sensors, memory, number of individuals it can sample, and energy constraints [8]. This impacts the number of measurements that can be taken. Therefore, it is of great importance to calibrate the algorithms that measure the anomalous behavior exactly right. However, there is a fundamental trade-off that is important to consider: at the core, a more sensitive algorithm may detect (almost) all anomalous behaviors (true positives), but will also detect some non-anomalous behaviors as anomalous (false positives). Reducing the sensitivity will decrease the rate of false positives, and simultaneously increase the rate of correctly identified negatives (true negatives). Consequently, this will also increase the number of cases where anomalous behaviors are not detected as such (false negatives). False positives and false negatives are also associated with so-called type I and type II errors, respectively [20]. Table 1 summarizes the four cases and relates them to our concrete application.

The trade-off between true positives and false positives is a fundamental problem of classifying data into binary categories, plaguing all diagnostic fields [21,22,23] and many organisms. For example, in the context of antipredator vigilance, false alarms “have been documented in several species and represent a surprisingly high proportion of all alarms” [24] (p. 1199). Resulting alarm calls and escape behavior cost valuable energy and thus should be minimized. However, the fact that false alarms often have lower costs than missing a threat may bias the perception systems of organisms towards the former, even though it comes at a cost of overall accuracy [25,26]. This fact helps to explain simple behaviors such as animals that “retreat quickly to protective cover, sometimes even in the absence of any obvious source of danger” [27] (p. 1563). Similar tendencies to be highly sensitive towards threats seem to be present in humans as well. It has been shown that participants reliably overestimate the change in level of rising level tones [28]. This could provide a selective advantage by indicating that the sound source is closer than it actually is and cause an earlier defensive response [29]. The same bias towards true positives—and that towards false positives—can be beneficial for organisms not only when it comes to detecting threat, but also when it comes to detecting desirable outcomes. For example, it has been suggested that human males have a “sexual overperception bias” because the costs of failing to notice a potential partner are higher than mistakenly assuming that someone is not one [30,31]. These examples illustrate that accuracy is signal detection is important, but that signal detection might be skewed towards high sensitivity because missing certain signals can have dire consequences. Similarly, missing out on acting on an environmental catastrophe seems to be more consequential than taking measures towards a healthy system. In line with this and the precautionary principle [32], we assume that for our use case, false negatives are more costly than false positives.

Because it is unfeasible to construct the spinning detection algorithm in a way so that it does not exhibit any false positives or false negatives at all, other mechanisms to improve the accuracy would be useful. One way to improve the accuracy is to check for external factors. For example, sniffer dogs may have false negative responses, but have longer sniffing times in such cases than when they have true negative responses [33]. Analogously, the biohybrid may have longer processing times when detecting false negatives as compared to true negatives. However, because the biohybrid is supposed to operate autonomously, no other authority can use such factors to correct the classifications of the biohybrid. Because of that, it is also unfeasible to make the biohybrid learn how to improve the classification of spinning behavior of *Daphnia* once it is in the field: the lack of feedback about the classifications rules out (deep) reinforcement learning [34] and related mechanisms. A more trivial way to increase the accuracy would be to increase the number of measurements. That multi-step measurement systems can improve accuracy has been shown also in the related context of water level monitoring [35]. However, multi-step measuring would need additional recordings and computing time, which is a problem for biohybrids that are limited in their battery and memory capacities.

The core principle that we propose is as follows: a number of different estimates are combined to an aggregate estimate. That additional opinions can improve estimates has been shown in a variety of contexts [36,37]. In many of those contexts, the mean estimate of a group of raters comes close to the true value, although there are examples where the same procedure does not work [38]. Aggregating measurements of multiple independent sensors is often referred to as “sensor fusion” [39]. However, such algorithms are mainly applied in swarm robotics, where other members are available and information from different sensors can be pooled. However, the biohybrid operates alone, making ideas based on swarm intelligence, group behavior or quorum sensing unfeasible. This limitation is what makes our method relevant: We propose a method to improve signal detection using a consensus based algorithm, which does not need other individuals. With our proposed method, the biohybrid becomes its own committee.

**Figure 2:**
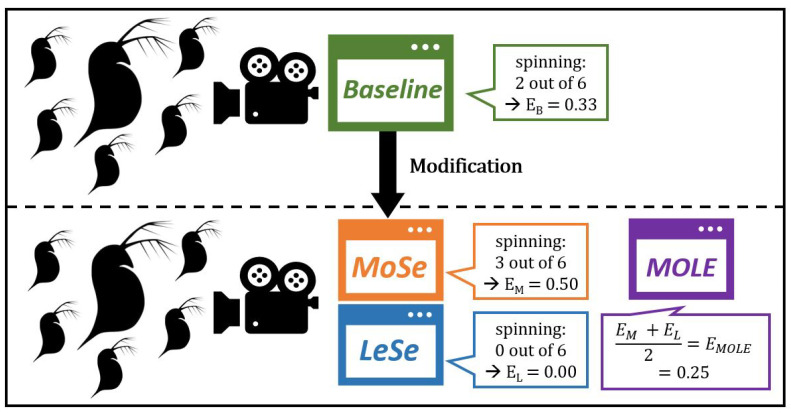
An existing baseline algorithm B gets marginally modified to produce a more sensitive algorithm (MoSe) and a less sensitive algorithm (LeSe). The MOSE algorithm is a meta algorithm that combines the two estimates into one to achieve higher accuracy.

Usually, aggregation of opinions improves the accuracy of estimations because more data is integrated into the estimations. However, because of energy and memory constraints, the biohybrid cannot collect extensive amounts of data. We hypothesize that it could be possible to modify an existing baseline image analysis algorithm (Baseline) so that it is a bit more sensitive. In addition to this modified algorithm (MoSe), one could modify the Baseline algorithm to create a less sensitive algorithm (LeSe). The estimation of the MoSe algorithm, together with the estimation of the LeSe algorithm, can then be used to produce a combined estimate by averaging the two estimations. We call this averaging algorithm MOLE because it combines estimates of an algorithm that is more sensitive and of one that is less sensitive than the unmodified baseline algorithm. It is possible to further enhance the accuracy of such combined judgements by weighting the different opinions that are involved [40,41]. However, since we only have two algorithms and little knowledge about which of them performs better, we use straight averaging. We show this principle schematically in Figure 2.

By using the MOLE algorithm, the biohybrid can obtain a “second opinion” from itself. Extensive testing of the method we propose is necessary, since it is not obvious that socially acquired information is always beneficial [27]. We created and applied a mathematical model that describes our task and solve it by simulation.

## 2. Materials and Methods

### 2.1. Task Description and Model Implementation

We used computational simulation to estimate the expected accuracy of the biohybdrid’s estimations using different algorithms. In the following, we first describe the task, how we modeled it, and the metrics used to evaluate performance. We then describe the different algorithms and parameters that we modify in the experiments. We conclude this section by describing the experiments that we conducted.

One of the tasks of the biohybrid is to detect the spinning behavior of *Daphnia* in a lake (or a certain area within it) and estimate how common this behavior is. To sample all or most *Daphnia* of a given area in the lake is neither desirable, since it would likely disturb the sampled individuals, nor feasible, since *Daphnia* populations consist of thousands of individuals. Instead, the proposed biohybrid relies on a small sample of a few individual *Daphnia*. In this study, we assume that the biohybrid has a sample of 6 individual *Daphnia* in the observation chamber, which are used as a sample to estimate the prevalence of spinning behavior in the population. For example, if 0 out of 6 *Daphnia* in the sample are spinning, it will be reasonable to estimate that the population proportion will be close to 0. If 3 out of 6 *Daphnia* are spinning, the population proportion will likely be closer to 0.5. We make an important consideration: any algorithm used by the module will produce some amount of false positive and false negatives. This complicates the task. In short, the task is to estimate the population proportion under uncertainty, using a relatively small sample.

In the following, we use capital letters to distinguish the metrics from the common meaning of the words, e.g., “Estimate” denotes the estimate of the population proportion produced by an algorithm, while “estimate” has its usual meaning. Further, we use italics for the names of the algorithms to highlight them in the text.

We assume that the *Daphnia* in the observational chamber behave indistinguishable close to the *Daphnia* in the lake. Therefore, observing the six *Daphnia* in the chamber is analogous to drawing six samples from a population. We modeled the *Daphnia* population as a stream of *N* (10,000) binary signals, consisting of 0 s and 1 s in a random order. In this signal stream, a certain proportion of signals will be 1, each one representing a spinning individual of *Daphnia* in the population. Accordingly, we denote this population proportion as *P*, and it is calculated simply as

(1) $$P=\frac{N_{1}}{N_{0}+N_{1}}$$

where $N_{0}$ and $N_{1}$ represent the absolute number of 0-signals and 1-signals, respectively. The task consists of estimating *P* given a sample of the size S. This means, that the algorithms will produce an estimate after *S* presented signals. The signals presented are either 0 or 1, and will be perceived with the assumed rates by a Baseline algorithm shown in Table 2.

If the algorithm measures a signal to be 0, it will increase the $M_{0}$ counter, and it will do the same for signals that are measured to be 1 and the $M_{1}$ counter. Consequently, what the Baseline algorithm has measured might deviate from the actual signals that were presented. After S signals, the Baseline algorithm produces an estimate defined as

(2) $$E=\frac{M_{1}}{M_{0}+M_{1}}$$

which, because no signals are ever completely missed in our model, is equivalent to the number of signals detected to be 1, divided by the sample size *S*

(3) $$E=\frac{M_{1}}{S}.$$

We measure the success of estimating *P* by comparing the estimate *E* of the Baseline algorithm to *P*. For a well-performing algorithm, it holds that

(4) E≊P.

Further, we assume that overestimating *P* is as bad for the Accuracy as underestimating *P*, which is why we measure the Accuracy *A* as

(5) A=1−|P−E|

and the closer the Accuracy of an algorithm is to 1, the better it performs. To measure how much gain in Accuracy is achieved in relation to the performance of the Baseline algorithm, we define the “Advantage” of algorithms as

(6) $$ADV=\frac{A-A_{B}}{A_{B}}$$

where *A* is the accuracy of the algorithm in question, and $A_{B}$ is the accuracy of the Baseline algorithm that serves as a benchmark. The resulting Advantage represents the relative gain in Accuracy. For example, if the Baseline algorithm has an Accuracy of 0.4, and another algorithm has an Accuracy of 0.6, then the Advantage is 0.5. However, when the Accuracy of the Baseline algorithm is at an already high 0.8, and another algorithm has an accuracy of 1.0, then the Advantage is less substantial, being 0.25, even though the absolute difference in Accuracy is the same in both cases. Notably, the Advantage of a tested algorithm can also be negative, which implies that the Baseline algorithm outperforms the tested algorithm instead of vice versa. Multiplying the Advantage value by 100 indicates the increase in Accuracy in percent. For example, an algorithm having an Advantage value of 0.1 implies that its Accuracy is 10% higher than the Accuracy of the Baseline algorithm.

### 2.2. Description of Algorithms Used in the Study

We consider two algorithms that are based on the Baseline algorithm. A more sensitive algorithm MoSe, as well as a less sensitive algorithm LeSe have slight alterations expressed as Delta (Δ) in their signal detection matrices, shown in Table 3 and Table 4 respectively.

Aside from the differences in the probabilities to produce TP, FP, FN, and TN cases, the algorithms function the same as the Baseline algorithm. They sample a number of S signals, increase their $M_{0}$- and $M_{1}$-counters and then produce an estimate based on that according to Equation (3). In order to have a control group, we also analyze a random algorithm (Random) that produces random uniformly distributed floating numbers between 0 and 1 as estimates for P.

The main novelty that we propose is that one could combine the estimates of two algorithms that are based on the Baseline algorithm into one estimate, and potentially see an increase in accuracy. To test this idea, we proposed an algorithm that does exactly that. The “More and Less” algorithm MOLE combines the estimates from MoSe and LeSe. The estimates are combined simply by producing the arithmetic average of the estimates involved. Table 5 summarizes all hypothetical algorithms that are used and get compared in the study.

### 2.3. Conducted Experiments

In this section we describe the experiments that we conducted. To analyze and compare the described algorithms comprehensively, we adjusted and modified some of their properties. These properties and their adjustments are explained in Table 6. We use capital letters to distinguish the properties from the common meaning of the words, e.g., “Quality” denotes the property of the Baseline algorithm, while “quality” has its usual meaning.

We show in Table 6 the ways in which our algorithms can be modified. The Baseline algorithm can be imprecise and have many false positives and false negatives (low Quality) or it can have few of those misclassifications (high Quality). The Baseline algorithm may also be biased towards a high number of true positives and have a lower number of true negatives (high Sensitivity Bias), or vice versa (low Sensitivity Bias). This is only considered in experiment 3, and in all other experiments the Sensitivity Bias is 0, meaning that the rate for true positives is equal to the rate of true negatives. The algorithms MoSe and LeSe have a parameter called Delta. This determines the deviation from the TP-rates and the TN-rates of Baseline. We refer to Table 3 and Table 4 for examples. The two algorithms also have a parameter called Handicap, which is used to decrease their quality. For example, if MoSe has a TP-rate of 0.8 and a TN-rate of 0.6, then a Handicap of 0.1 would reduce these rates to 0.7 and 0.5, respectively. Lastly, we use the parameter Asymmetry to modify the MOLE algorithm. Essentially, it determines which variants of the MoSe and LeSe algorithm are used to produce the combined estimate. If Asymmetry is 0, then MoSe and LeSe have the same Delta. If Asymmetry is 1, then MoSe has the indicated Delta, while LeSe has a Delta of 0. If Asymmetry is −0.7, then LeSe has 70% of the indicated Delta, and MoSe has 30% of the indicated Delta. With that, the Asymmetry parameter regulates in which direction and to which extend MOLE is biased.

With the exception of two experiments (experiment 5 and experiment 6), we always simulated two scenarios. In scenario 1, the population proportion of spinning *Daphnia* is a randomly generated uniformly distributed floating number between 0 and 1. In scenario 2, we assume that the population proportion of spinning *Daphnia* is normally distributed around 0.1 with a standard deviation of 0.025.

There are two experiments where we do not compare 2 scenarios. In experiment 5, we test different values for the population proportion P. In experiment 6, we simulate a monitoring period consisting of two measurements. There, we simulate the diagnosis of a potential “catastrophic event” within in the lifetime of the biohybrid. For this, the rate of spinning *Daphnia* in the population (P) is a random floating number between 0 and 1. The biohybrid measures as in the other experiments, but it measures twice. For the first measurement, the biohybrid assesses 6 *Daphnia*. We simulate the case of 2 *Daphnia* dying, which only leaves 4 for the second measurement. Because it is not exactly known which population proportion of spinning *Daphnia* constitute a catastrophe, we simulate two cases. In the first case, we consider the situation as “catastrophic” if P is above 66%. If the biohybrid detects more than 40% spinning *Daphnia* twice in a row, it sets an alarm. In the second case, we consider the situation as “catastrophic” if P is above 30% and the biohybrid sends an alarm if the biohybrid detects more than 25% spinning *Daphnia* twice in a row. When the biohybrid sends an alarm when P > 66% (or P > 30% in the second case), we consider this a true positive; if it fails to do so, we consider it a false negative. If the biohybrid sends an alarm when P < 66% (or P < 30% in the second case), we consider this a false positive, and if it does not, we consider it a true negative. We are interested in the performance of the algorithms as well as in the number of false negatives, which we consider a more serious error than false positives. Ideally, an algorithm detects more true positives and true negatives and has a low false negative rate.

For experiment 1, we are interested in the influence of the Quality on the Accuracy of the Baseline algorithm. The results obtained from this experiments help to indicate the necessary Quality for the Baseline algorithm if a certain target Accuracy should be reached. We perform 10,000 runs for each parameter setting and report the mean results.

For experiment 2, we compare the Baseline algorithm and the MOLE algorithm to see if there is a benefit to be expected when using MOLE. We hypothesize that the MOLE algorithm is especially beneficial when the Quality of the Baseline algorithm is low. We perform 1000 runs for each parameter setting and report the mean results.

For experiment 3, we compared the MOLE algorithm to modified Baseline algorithms. We hypothesize that the advantage gained by applying the MOLE algorithm is dependent on the “Sensitivity Bias” of the Baseline algorithm. We perform 10,000 runs for each parameter setting and report the mean results.

For experiment 4, we wanted to see if suboptimal implementations of the MOLE algorithm can outperform the Baseline algorithm. For this, we used a Handicap to reduce the classification accuracy of the MoSe and LeSe algorithms (which are the foundation of the MOLE algorithm). We perform 10,000 runs for each parameter setting and report the mean results.

For experiment 5, we analyze if it is fundamental for the MOLE algorithm to be symmetrical, i.e., to consist of MoSe and LeSe algorithms that deviate equally from the Baseline algorithms. We measure the Advantage with set population proportions. We perform 2500 runs for each parameter setting and report the mean results.

For experiment 6, we simulate the diagnosis of a potential “catastrophic event” within in the lifetime of the biohybrid, as described above. We measure true positives and true negatives, as well as false positives and false negatives. We perform 10,000 runs for each parameter setting and report the mean results.

## 3. Results

We study the degree of accuracy that can be reached by using a small sample of 6 individuals to estimate the population proportion under the consideration of potential false positives and false negatives.

**Figure 3:**
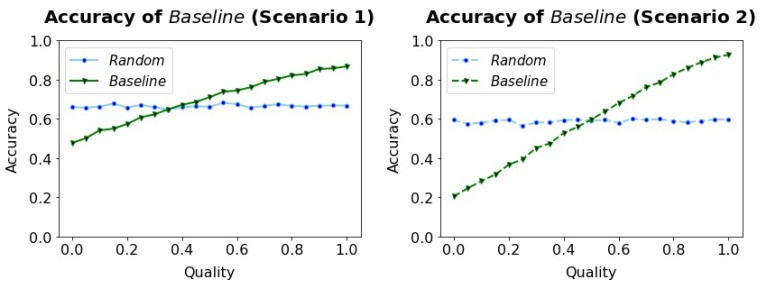
In both scenarios, the Accuracy of the Baseline algorithm increases with increasing Quality. Even with a perfect algorithm (Quality = 1), 100% Accuracy cannot be reached.

We found that the Baseline algorithm outperforms the Random algorithm if the Quality surpasses 0.5 in both scenarios, see Figure 3. A target Accuracy of around 0.8 can be reached with a sensor of Quality 0.7 in both scenarios.

**Figure 4:**
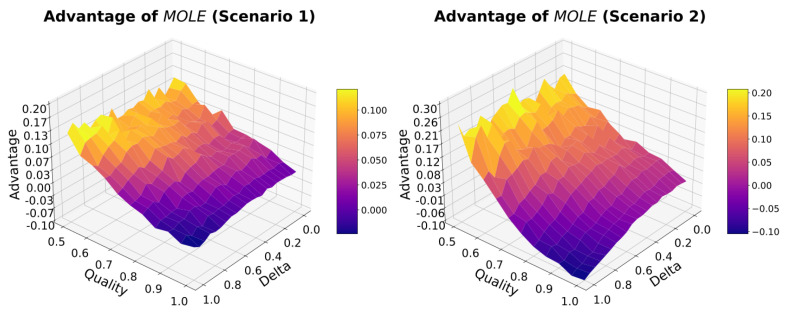
There is an Advantage in using the MOLE algorithm in many cases. Only when the Quality of the Baseline algorithm and Delta are high simultaneously, the Baseline algorithm outperforms the MOLE algorithm.

To answer if the method of combining estimates of two algorithms would improve the Accuracy, we measure the Advantage of MOLE. For this, we perform a parameter sweep over the parameters Quality and Delta, see Figure 4.

We found positive values for the Advantage of MOLE over Baseline, which suggests a benefit of using the former algorithm over the latter. Especially in cases in which the Quality of Baseline is low, the Advantage is high. We found a parameter space in which there is a negative advantage when using MOLE in the cases where both Quality and Delta are high (0.8 and higher).

**Figure 5:**
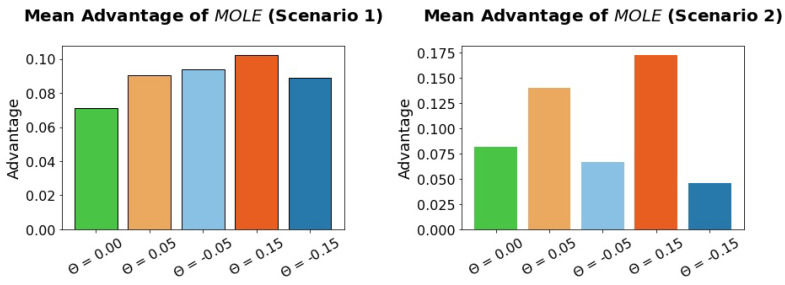
The MOLE algorithm outperforms different variants of the Baseline algorithm in both scenarios. If the Baseline algorithm is biased towards high sensitivity (orange bars), MOLE has especially high Advantage values in scenario 2. There is also an Advantage when the Baseline algorithm is biased towards high specificity (blue bars).

We compared MOLE algorithm with different variants of the Baseline algorithm, i.e., variants that either have higher sensitivity than specificity (Θ > 0.0) or vice versa (Θ < 0.0). To see if there are scenarios were the Advantage gained by using MOLE is especially high or low, we measured the advantage of MOLE over different variants of the Baseline algorithm, see Figure 5.

**Figure 6:**
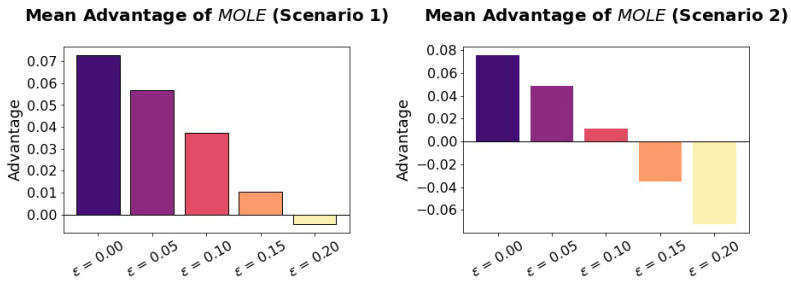
The Advantage of the MOLE algorithm over the Baseline algorithm becomes less with increasing Handicap (ϵ). Up to ϵ = 0.10, MOLE has an significant Advantage in scenario 1, while this is only true until ϵ = 0.05 in scenario 2.

To see if the MOLE algorithm has an advantage if it has a Handicap (ϵ > 0.0), we compare it to the Baseline algorithm under varying amounts of Handicap, see Figure 6.

Increasing Handicap decreases the Advantage of MOLE. For each scenario, there is a turning point at which point MOLE has a negative Advantage over Baseline, which means it is outperformed by Baseline. This turning point is around ϵ = 0.20 in scenario 1, and around ϵ = 0.15 in scenario 2.

**Figure 7:**
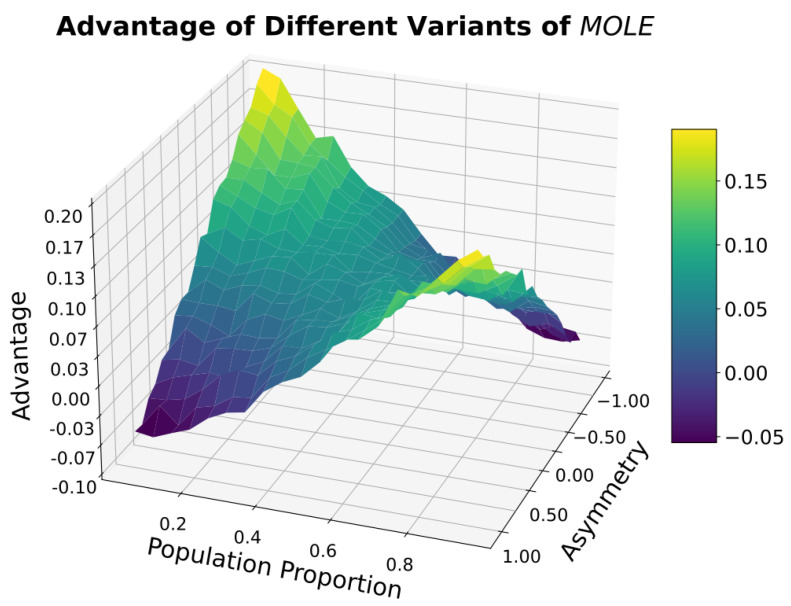
The Advantage of the MOLE algorithm over the Baseline algorithm is influenced by the Asymmetry, and this influence becomes especially salient when P is close to 0 or close to 1.

There is an interaction between the population proportion P and the Asymmetry of the MOLE algorithm. We found an advantage of the MOLE algorithm within most of the parameter space, see Figure 7. In cases where the population Proportion is low (below 0.2) and the Asymmetry value is simultaneously high (above 0.5), as well as in the converse case, there is a negative Advantage of using the MOLE algorithm.

**Figure 8:**
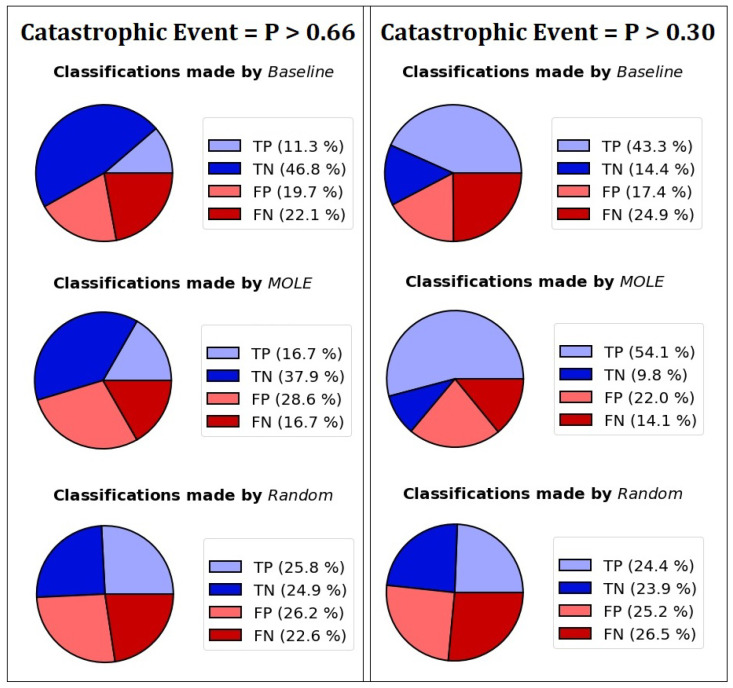
The Baseline algorithm and the MOLE algorithm have different rates of classifications. Correct classifications (TP and TN) are shown in blue, and misclassifications are shown in red (FP and FN).

We were interested if Baseline and MOLE have similar capabilities of diagnosing a simulated catastrophic event, and show the results of our experiments in Figure 8. Both algorithms have a similar percentage of correct classifications (TP+TN), but differ in their probabilities to send false alarms (FP) and to miss catastrophic events (FN). We found that the MOLE algorithm has a significantly lower proportion of false negatives.

## 4. Discussion

In this study, we simulated a biohybrid that uses an image analysis algorithm to estimate the population proportion of spinning *Daphnia*. We found that for this task, even with a small sample of 6 individuals and an imperfect spinning detection algorithm, acceptable accuracy of 80% or higher can be reached. Further, we studied if the accuracy of these estimations can be improved by modifying the algorithm to be more sensitive and combining the estimations of the modified algorithm with another algorithm that has been modified to be less sensitive. We found that this method can increase the accuracy by up to 20% in some cases. Lastly, we found that our proposed method has markedly different rates of false positives and false negatives. Notably, the false-negative rates were lower, which we consider beneficial for practical application in the context of environmental monitoring.

The fact that additional opinions can improve estimations is not new and has been, for example, well documented in the domain of forecasting [42]. However, in our study we showed that it is not necessary to have a high number of different opinions to gain an advantage, and that the estimation of the population proportion is a task where this method is well applicable. This is an important finding that has not been discussed in the context of environmental monitoring using biohybrids. Surprisingly, we also found an increase in accuracy when modifying the MoSe and LeSe algorithms to have lower quality compared to the Baseline algorithm in experiment 4, see Figure 6. In these cases, two algorithms, which would perform worse on their own, have better combined estimates then a well performing algorithm. A “less skilled”, yet diverse group, outperforming high performers has also been shown in the context of problem-solving [43], but it was not shown before that this approach can be used for environmental monitoring. These results are not explained by the Condorcet Jury Theorem [44], as it only applies to binary choice problems, while this task requires an estimation of a rate. Neither is the high performance of the MOLE algorithm better explained by the many-wrongs principle [45] nor is it necessary for the biohybrid to apply advanced ratio and difference estimators [46,47]. Instead, one explanation for the high performance of the MOLE algorithm is related to the limited sample size. It was shown that the MOLE algorithm increases the accuracy of the detection levels by both over and underestimating the rates that would be normally estimated based on the observed behavior. For example, a scenario where 42% of *Daphnia* are stressed in a habitat is unlikely to be detected accurately with the narrowly limited sample size present in the biohybrid. By applying the MOLE algorithm, the biohybrid can average the under (i.e., 33%) and overestimations (i.e., 50%) of the observed number of stressed individuals and thus, produce results as close to the real scenario as possible (41.67%). Further, we found that it is not necessary for this under- and overestimations to be symmetrical. We show in experiment 5 that also lopsided variants of the MOLE algorithm outperform the Baseline algorithm in most scenarios, see Figure 5.

Currently, the algorithm for *Daphnia* detection is under development in the EU project Robocoenosis [8]. This ongoing research focuses on improving object-detection and object-tracking methods. For the purpose of this paper, the numbers of spinning *Daphnia* were chosen as examples to show the power of the presented algorithm. They are based both on the literature search and in-house experiments (data not shown). They provide an estimate of real-world conditions, but are subject to change, should more research be carried out for the usage of *Daphnia* for long-term observations in the biohybrid module. In such cases, it is possible to adjust the threshold classifying the group as “stressed” (i.e., the minimum number of spinning individuals). This higher sensitivity will come with lower specificity, however, it is possible to account for it by developing another algorithm (highly specific and less sensitive). From there, we can create a series of sister algorithms which, working together, provide the best estimation of the ecological status of the lake.

In addition to the MOLE algorithm providing a more accurate interpretation of the collected data, it requires a relatively small amount of additional effort to implement. The Baseline algorithm is a necessity for the proper functioning of the biohybrid. The time and effort input is comparatively small, as it is merely an addition to an already existing system. Even if the implementation of the MOLE algorithm is imperfect, e.g., because the MoSe and the LeSe algorithm individually perform worse than the Baseline algorithm, a significant advantage using the MOLE algorithm is to be expected, see Figure 7. It offers the possibility of interpreting the state of the environment more accurately without making changes to the methodology used for constructing the biohybrid.

Additionally, the biohybrid entity developed by Robocoenosis, which is here used as a case study, aims at incorporating multiple lifeforms. The machine will read the stress levels from individual modules (such as the *Daphnia* module) and base its overall result on their collective responses. Other organisms taken under consideration for this methodology are *Hydra* sp., bivalves (especially zebra mussel *Dreissena polymorpha*) and plankton community structure. The MOLE algorithm can be used for those analysis in the future to optimize the amount of useful information we can obtain from both individual modules as well as the entire biohybrid entity.

## 5. Conclusions

The accuracy of estimations is often improved if additional opinions are considered. We showed that this principle of the “wisdom of the crowd” [48] can be applied in our use case. This use case was the estimation of the population rate of spinning *Daphnia* using a limited sample size. The proposed biohybrid can obtain a “second opinion” from itself by analyzing the recorded images of *Daphnia* in two different ways and averaging the estimations. We found in simulation that this approach requires relatively little effort and provides gains in accuracy for a biohybrid at its quest of environmental monitoring.

## Figures and Tables

**Table 1:** Possible cases of *Daphnia* behavior detection.

Behavior	Detected as	Case
Spinning	Spinning	True positive (TP)
Not spinning	Spinning	False positive (FP)
Spinning	Not spinning	False negative (FN)
Not spinning	Not spinning	True negative (TN)

**Table 2:** Signal-detection matrix of the Baseline algorithm.

True Value of Signal	Probability to Detect It as 0	Probability to Detect It as 1
0	0.75 (TN-rate)	0.25 (FP-rate)
1	0.25 (FN-rate)	0.75 (TP-rate)

**Table 3:** Signal-detection matrix of the more sensitive algorithm MoSe.

True Value of Signal	Probability to Detect It as 0	Probability to Detect It as 1
0	0.75 − Δ (TN-rate)	0.25 + Δ (FP-rate)
1	0.25 − Δ (FN-rate)	0.75 + Δ (TP-rate)

**Table 4:** Signal detection matrix of the less sensitive algorithm LeSe.

True Value of Signal	Probability to Detected It as 0	Probability to Detected It as 1
0	0.75 + Δ (TN-rate)	0.25 − Δ (FP-rate)
1	0.25 + Δ (FN-rate)	0.75 − Δ (TP-rate)

**Table 5:** Algorithms used in this study.

Name	Description
Baseline	Baseline algorithm where the TP-rate is the same as the TN-rate, unless modified by a Sensitivity Bias (Θ) ≠ 0.
MoSe	Algorithm that, compared to Baseline, has a higher TP and FP-rate, but a lower TN-rate and FN-rate. The difference is Delta (Δ).
LeSe	Algorithm that, compared to Baseline, has a lower TP-rate and FP-rate, but a higher TN-rate and FN-rate. The difference is Delta (Δ).
Random	Algorithm that produces a uniformly distributed random floating number between 0 and 1 as an estimate.
MOLE	Algorithm that combines estimates of MoSe and LeSe by averaging arithmetically.

**Table 6:** Properties of algorithms that get modified for experiments.

Name (Abbreviation)	Owner	Standard Value	Description
Quality (Q)	Baseline	0.75	Determines the base TP-rate and the TN-rate (changed in experiment 1 and 2).
Delta (Δ)	MoSe and LeSe	0.10	Determines deviations of detection rates from Quality (changed in experiment 2).
Sensitivity Bias (Θ)	Baseline	0.00	Determines if TP-rate=TN-rate or if there is a bias towards either higher sensitivity or higher specificity (changed in experiment 3).
Handicap (ϵ)	MoSe and LeSe	0.00	Determines the decrease in TP-rate and TN-rate (changed in experiment 4).
Asymmetry	MOLE	0.00	Determines if MoSe and LeSe deviate equally from Baseline (changed in experiment 5).

## Data Availability

Data is available upon request.

## Abbreviations

The following abbreviations are used in this manuscript:

MoSe	Name of a modified Baseline algorithm that is more sensitive
LeSe	Name of a modified Baseline algorithm that is less sensitive
MOLE	Name of an algorithm that combines the estimates of MoSe and LeSe
